# Attitudes to incorporating genomic risk assessments into population screening programs: the importance of purpose, context and deliberation

**DOI:** 10.1186/s12920-016-0186-5

**Published:** 2016-05-23

**Authors:** Stuart G. Nicholls, Holly Etchegary, June C. Carroll, David Castle, Louise Lemyre, Beth K. Potter, Samantha Craigie, Brenda J. Wilson, On behalf of the CIHR Emerging Team in Genomics and Screening

**Affiliations:** School of Epidemiology, Public Health and Preventive Medicine, University of Ottawa, Ottawa, ON K1H 8M5 Canada; Clinical Epidemiology, Memorial University, St John’s, NL Canada; Department of Family and Community Medicine, Sinai Health System, University of Toronto, Toronto, ON Canada; Sydney G. Frankfort Chair in Family Medicine, Toronto, ON Canada; Vice-President Research, University of Victoria, Victoria, BC Canada; School of Psychology, University of Ottawa, Ottawa, ON Canada; Michael G DeGroote National Pain Centre, McMaster University, Hamilton, ON Canada

## Abstract

**Background:**

The use of an overall risk assessment based on genomic information is consistent with precision medicine. Despite the enthusiasm, there is a need for public engagement on the appropriate use of such emerging technologies in order to frame meaningful evaluations of utility, including the practical implementation and acceptability issues that might emerge. Doing so requires the involvement of the end users of these services, including patients, and sections of the public who are the target group for population based screening. In the present study we sought to explore public attitudes to the potential integration of personal genomic profiling within existing population screening programs; and to explore the evolution of these attitudes as part of a deliberative process.

**Methods:**

We conducted a mixed methods study presented in the format of a deliberative workshop. Participants were drawn from communities in Ottawa, Ontario (ON) and St John’s, Newfoundland and Labrador (NL), Canada. Individuals were approached to take part in a workshop on the incorporation of genomic risk profiling for either colorectal cancer screening (CRC), or newborn screening for type 1 diabetes mellitus (T1DM).

**Results:**

A total of *N* = 148 (*N* = 65 ON, *N* = 83 NL) participants provided data for analysis. Participants in both groups were supportive of public funding for genomic risk profiling, although participants in the T1DM groups expressed more guarded positive attitudes than participants in the CRC groups. These views were stable throughout the workshop (CRC, *p* = 0.15, T1DM, *p* =0.39). Participants were less positive about individual testing, with a significant decrease in support over the course of the workshop (CRC *p* = 0.02, T1DM, *p* = 0.003). Common concerns related to access to test results by third parties.

**Conclusions:**

The findings of this study suggest that members of the target populations for potential genomic profiling tests (designed for screening or risk prediction purposes) can engage in meaningful deliberation about their general acceptability and personal utility. Evaluations of whether a test would be personally useful may depend on the experience of the participants in personal health decision making, the purpose of the test, and the availability of interventions to reduce disease risk.

**Electronic supplementary material:**

The online version of this article (doi:10.1186/s12920-016-0186-5) contains supplementary material, which is available to authorized users.

## Background

Rapid advances in technology since the completion of the Human Genome Project have led to significantly decreased costs and timeframes for integrating genomic information into routine health care [1]. While much attention has been given to the potential of whole genome or exome sequencing, considerable efforts have also been directed towards more targeted approaches. One of these is genomic profiling, in which multiple common genomic susceptibility variants are combined to offer risk stratification for a target disease [2]. Genomic profiling is distinguished from genetic testing by its focus on normal variation, modest risk alterations, and an integrated approach, rather than on detecting specific, rare disease-associated, high penetrance alleles [3]. In particular it involves examining the genome for single nucleotide polymorphisms (SNPs) [3], the most common type of genetic variation related to common disease [4, 5]. While each variant may be only weakly associated with pathology, it has been proposed that a combination of variants may collectively explain a portion of the risk of a given disease [6].

While the predictive ability of such assessments may be limited when applied to individuals, and will vary between conditions due to the genetic complexity of the disease in question and influence of the environment on gene expression [7, 8], incorporating genomic profiling data in population based screening programs – in addition to risk based primarily on age, for example – could reduce false positive or negative results, and over-diagnosis [2, 9]. If successful, this would reduce harms such as anxiety and unnecessary diagnostic tests, and improve the use of scarce health care resources.

While there is much expressed enthusiasm about the idea of personalized medicine and ‘personal genomics’, there is a need for public engagement on the appropriate use of such emerging technologies in reality. We need to know more about how they might play out in practice in order to frame meaningful evaluations of utility, including the practical implementation and acceptability issues that might emerge. For example, to what extent do traditional principles of screening such as actionability of results weigh into participants’ evaluations and intentions [9] compared with emergent issues such as ‘personal utility’ [9–11] or characteristics of the test or disease in question [12, 13]? These questions require the involvement of those who are the end users of these services, including patients, and sections of the public who are the target group for population based screening.

### Engaging with the public about genomics

The engagement of publics in policy discussions about topics such as genomics and healthcare can be justified on a number of grounds. Firstly, legitimacy and fairness demand that in a healthcare system that is publicly funded, the public be involved in decisions regarding how those funds are used. Greater involvement of the public is also in keeping with democratic principles [14], may provide greater support for the decisions made (assuming they are compatible with the expressed views), and could potentially lead to increased trust in decision-makers and the decision-making process.

Beyond the potential impact on individuals’ anxiety or motivation, augmenting risk information through genomic profiling might eventually change implicit frameworks of understanding about disease causation [15], the perceived role of prevention and lifestyle [16, 17], and the sense of personal responsibility for one’s own health [18, 19]. Concerns have also been raised about the privacy of personal genomic information, and that genomic risk information could be used in the calculation of, or eligibility for, individual insurance premiums or employers’ screening requirements [20–22]. While the Genetic Information Nondiscrimination Act (GINA) was signed into US law in 2008 [23], no such legislation exists in Canada, although this has been the subject of recent debate [24].

Finally, and to reiterate, the incorporation of public attitudes is a necessary component of implementation research: how members of target user groups view and respond to genomic information will influence acceptability and demand, and therefore set the upper limit on the overall beneficial impact that an effective technology will achieve in practice [1].

### The effect of deliberation on attitudes

The analysis of public attitudes is a developing area within the field of research focused on the integration of genomics into healthcare. A range of methods have been incorporated into existing studies, from traditional qualitative techniques such as focus groups, to quantitative approaches such as structured surveys [25–30]. More recently, approaches that seek to promote discussion and deliberation have been advocated, with the goal of obtaining more informed views and understanding of the issues at hand [31–35]. Such deliberative approaches tend to be characterized by their structure, often involving the provision of background information about the issue under discussion and relevant technical details, as well as their relatively small size and discursive format [31]. Implicit within this is the assumption that opinions will crystallize or even change as a result of the deliberative process [31, 36].

While we have previously explored the qualitative arguments provided by participants for their support for, or concerns regarding, the implementation of genomic risk profiles as part of population screening [37], there is a lack of data regarding the role that deliberation has on the formation of these attitudes. A recent review of public attitudes to genetics called “for more detailed research in particular aspects of attitudes about genetics in order to understand how these attitudes operate in behavior or policy preferences” [28], and some have called for research that investigates how much specific deliberative elements contribute to the outcomes of such exercises, given that there has been little research in this area [38].

The primary aim of this study was to examine the attitudes of specific population target groups to the potential integration of hypothetical genomic profiling, using their experiences with existing screening programs as a starting point for deliberation. Secondary aims were to compare how the two groups responded, given the two different screening contexts, and to assess how much attitudes shifted during the deliberative process. As such, the study builds on previous research regarding public attitudes toward genomics to explore the influence of deliberative processes on attitudes and attempts to quantitatively evaluate the impact of context and process on the development of these attitudes.

## Methods

We conducted an exploratory, mixed methods study in which attitudes to hypothetical uses of genomic profiling were explored in semi-structured workshops. (Further details provided below.) We noted from previous studies that specific discussions of technologies may elicit attitude variations better than more abstract approaches. We therefore developed workshops on two topics to which we judged participants would be able to relate, and then recruited participants with demographic attributes to fit these notional screening approaches. We captured data in three ways: contemporaneous written comments by participants, structured survey items, and non-participant observation. Details of the study have been described previously [37]; we focus here on detailing the structured data collection and analysis.

### Topic selection

The two workshop topics were genomic profiling for colorectal cancer (CRC) risk assessment in adults, and for type 1 diabetes (T1DM) risk assessment in infants. CRC is a leading cause of mortality in North America [38, 39], with an inherited component to its incidence [40, 41]. Evidence supports the potential for genomic profiling based on multiple common variants as an approach to risk stratification, and possibly incorporation into population screening [6, 40, 42]. T1DM is a chronic disease most often diagnosed in childhood [43, 44], and is apparently increasing in incidence [45]. The potential suitability of including T1DM in existing newborn screening (NBS) panels has been discussed [46, 47].

### Deliberative workshop format

**Fig. 1:**
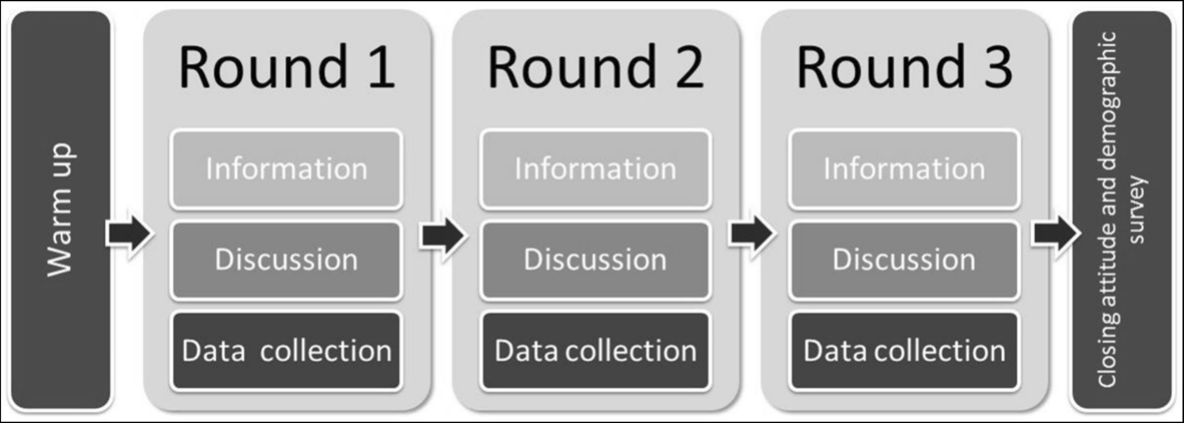
Schematic of the workshop format

The deliberative workshop approach was informed by previous public engagement studies examining socio-ethical issues in genomics [48–50]. A summary of the approach is presented in Fig. 1. Briefly, each workshop comprised three rounds, each of which itself contained three components: a standardized information set relating to the hypothetical genomic risk profiling test under discussion, a deliberative period for plenary discussion, and brief structured collection of attitude data.

The details of the information sets are provided in Table 1 (see also Additional file 1). They were developed by a multidisciplinary group which included clinical experts, health psychologists, and epidemiologists. The set for each workshop comprised three parts, with the content moving from (a) basic orientation to the clinical context, including the rationale for the use of genomic test, to (b) consideration of potential individual benefits, harms, and consequences, to (c) consideration of wider issues including ethical and social issues. They therefore included background information on current (non-genomic) screening approaches, potential advantages and disadvantages of a genomic profiling approach, and potential impacts for themselves and family members. We emphasized that the tests were not yet available, and that their purpose was to inform risk assessment, but they were not themselves screening tests. In the absence of actual data on the validity or utility of these hypothetical tests, we extrapolated from evidence in the wider genetics literature. The information was worded neutrally and attempted to communicate a balanced approach to the potential merits or otherwise of the genomic tests. For the phrase “genomic profiling”, we substituted the term “DNA based risk test”, for two reasons: first, because the phrase “genomic profiling” is used in other contexts which might prove confusing to some participants (e.g., genomic profiling of tumours was not within the scope of this study), but we wished to retain a clear link to the idea of genetics or genomics; and second, because we wished to reinforce as much as possible the idea of linking the test with personal health risk in some way.

**Table 1:** Key elements of information sets

Information Set 1. The idea of genomic profiling	
• Description of colorectal cancer/newborn bloodspot screening program	
• Genomic profiling test (referred to as ‘DNA risk test’) and how this could be used implemented	
• Emphasis on the technology and improvement on current approaches, and potential benefits e.g. reducing unnecessary interventions and targeting interventions to those most at risk	
Information Set 2. The potential personal impacts of having a test	
• Potential advantages: lifestyle choices, screening participation, attending promptly to early symptoms; personal utility of knowledge irrespective of potential for risk reduction	
• Potential disadvantages: anxiety, depression, disease worry, reduced quality of life (if higher risk); failure to follow health advice, neglect of early symptoms (if lower risk)	
• Potential for effects on others: e.g. family members, as well access and use by third parties (e.g. insurance companies)	
• Idea that results are not transient but ‘for life’	
Information Set 3. Reiteration of the nature of such a test, and its place in personal health management	
• Integration of genomic profiling within broader set of risk assessment and screening tests	
• Reinforcement of *risk* not actual disease status conferred by the tests	

We used PowerPoint to present the information sets, and allowed questions for clarification. Deliberative components were discursive with the aim of discussing and debating the issues brought up by the information sets rather than *a priori* discussion questions. Participants were, however, not limited to discussing only the content of the information sets, allowing them to draw on previous topics or to raise new issues. The deliberative stages were facilitated by a member of the research team who also served as presenter for the information sets. The workshop finished with concluding structured attitude questions and collection of demographic data.

### Participants and recruitment

Participants were recruited from community based groups in Ottawa, Ontario (ON), and St John’s, Newfoundland and Labrador (NL), Canada. Participants were invited to take part in a workshop matched to their demographic characteristics.

Individuals were eligible for the CRC workshop if they were aged 50 years or older, consistent with eligibility criteria for provincial screening programs. Individuals were eligible for participation in the T1DM workshop if they had fairly recent experience of being offered NBS (defined in practice as a having child under five years of age). Hence, participants in the CRC workshops targeted older individuals, and the T1DM groups were younger.

In Ottawa, we recruited participants directly through community-based groups, such as seniors’ centres and groups for new parents. In St. John’s, individuals were recruited using random digit dialing. Irrespective of recruitment approach, participants were informed about the study and if they indicated interest they were provided written information and a consent form. On the day of the workshop participants were reminded of the study objectives and requirements and completed a consent form.

The protocol was approved by the Ottawa Hospital Research Ethics Board and the Newfoundland and Labrador Health Research Ethics Authority.

### Analytical strategy and data collection

As a mixed methods study, the three types of data collection were designed to be complementary and mutually informative. We report here the analysis of the structured data, reviewed in isolation from the other data sets. At the outset, it is important to note that, because the overall approach was qualitative, these analyses cannot be considered hypothesis testing, and the data themselves do not meet criteria for an epidemiological approach.

For the present analysis we focus principally on responses to the two “tracker” attitude questions that were repeated after each information set and discussion. We captured these responses repeatedly in order to assess how responses changed over the course of the workshop.

The first tracker question was designed to capture attitudes towards the general acceptability of the hypothetical test, and was worded:

*“If DNA risk tests for colon cancer/type 1 diabetes became available, do you think [Province] should pay for them?”*

The second tracker question was designed to capture the more personal assessment of the utility of the hypothetical test, and was worded:

*“When you think about your own/your child’s situation, would you want/want him or her to have a DNA risk test for colon cancer/type 1 diabetes?”*

For both questions, responses were coded on a 5-point Likert scale ranging from Definitely Yes to Definitely No.

We also present analyses of nine attitude questions included in the concluding survey (See Results and Table 2 for details). The items were developed to capture attitudes to issues framed at both policy/societal level (e.g. “I think that DNA risk tests for colon cancer are generally acceptable”), and personal level (e.g. “I would ask my doctor for this test”). Responses were scored as either Yes/No or on a 5 point Likert scale from Strongly Agree to Strongly Disagree. General feelings about the technology were captured by asking participants to select from a list of positive, negative and neutral “valence words” [51]. Standard demographic information was also collected.

**Table 2:** Demographic information of participants completing the survey measures

	CRC (N = 108) *n* (%)	T1DM (N = 40) *n* (%)	Fisher’s Exact test (2 sided)
Sex			*P* = 0.26
Female:	55 (23)	29 (73)	
Male	25 (51)	7 (17)	
Missing	28 (26)	4 (10)	
Marital Status			*P* < 0.01
Married or living with partner:	51 (47)	35 (87.5)	
Widowed	15 (14)	0	
Divorced or separated	10 (9)	0	
Single, never married	4 (4)	1 (2.5)	
Missing	28 (26)	4 (10)	
Highest level of educational attainment			*P* = 0.02
University degree - graduate or higher:	17 (16)	13 (33)	
University degree - undergraduate	15 (14)	9 (22.5)	
Community college, technical college, or CEGEP	23 (21)	8 (20)	
Secondary	15 (14)	1 (2)	
Professional degree	4 (4)	5 (12.5)	
Other training or education	6 (6)	0	
Missing	28 (26)	4 (10)	
Average income			*P* < 0.01
$0–29,999	13 (12)	1 (2.5)	
$30,000–49,999	17 (16)	1 (2.5)	
$50,000–69,999	18 (17)	6 (15)	
$70,000+	25 (23)	26 (65)	
Missing	35 (32)	6 (15)	
Ethnicity			*P* = 1.0
White	77 (71)	35 (87.5)	
Native Canadian	2 (2)	0	
Missing	29 (27)	5 (12.5)	
Language spoken at home			*P* = 0.38
English	73 (68)	35 (87.5)	
French	2 (2)	0	
Both English and French	5 (5)	0	
Missing	28 (26)	5 (12.5)	

*T1DM* Type 1 Diabetes Mellitus, *CRC* Colorectal Cancer, *CEGEP* Collège d’enseignement général et professionnel

### Analysis

All responses were collated and imported into IBM SPSS Statistics v22 [52] for analysis. Due to the relatively small sample sizes, purposive sampling, and exploratory nature of the study, extensive statistical analyses were inappropriate, and we took a largely descriptive approach. However, to assist interpretation of the comparisons within the workshop groups, and between the two topics, we selectively examined statistical significance with non-parametric tests.

For the tracker questions, we used the Friedman test, because of the skewness of the data. The Wilcoxon sign rank test was used to compare pairs of rounds, and The Mann Whitney *U* Test for comparisons of change in attitudes across groups. Responses to general attitude questions were compared using the *χ*^2^ statistic. For all statistical tests, a level of *p* <0.05 was accepted as significant.

## Results

Workshops were conducted between November 2011 and July 2012. A total of 200 participants attended the workshops (65 in ON, 135 in NL). Because of a technical problem, linked data for 52 NL participants are unavailable, so the current analysis reports on 148 participants. Of these, 108 participated in CRC workshops and 40 in NBS. Table 3 summarizes the demographic details of these samples.

**Table 3:** Responses to end of workshop questions

Item	CRC n/N (%)	TIDM n/N (%)	*P*-value (*Χ* ^2^)
DNA risk tests for condition generally acceptable	88/98 (90)	19/33 (58)	<0.01
Not interested in finding out risk of condition	8/97 (8)	13/33 (39)	<0.01
Concerned that high risk result would cause extra worry	46/97 (47)	28/33 (88)	<0.01
Concerned that test result would cause problems with insurance	71/96 (74)	30/33 (91)	0.24
Concerned about who might have access to test results	68/97 (70)	29/33 (88)	0.34
Knowing risk of condition would help plan properly for future	75/82 (87)	20/33 (69)	0.07
Test results could be important for other family members	84/97 (92)	22/32 (61)	<0.01

Percentage of respondents who Strongly agree or Agree, case study topic. % reported are valid percent based on responses*. CRC* Colorectal Cancer, *T1DM* Type 1 Diabetes Mellitus

Workshop sizes ranged from six to 35 participants. The mean ages of participants were 67 (range 53–88) years for CRC workshops and 35 (27–48) years for T1DM. Overall, participant groups were largely female (72 %), white (76 %), and English speaking (73 %). The average level of educational attainment was higher for ON participants than NL. The majority of respondents had not had personal experience of the condition in question (75/80 (94 %) in the CRC workshops, 39/40 (98 %) in the T1DM workshops), while almost a third of participants had a family member who had experience of the condition in question (26/79 (33 %) in the CRC workshops, 11/40 (28 %) in the T1DM workshops).

### Tracker questions

**Fig. 2:**
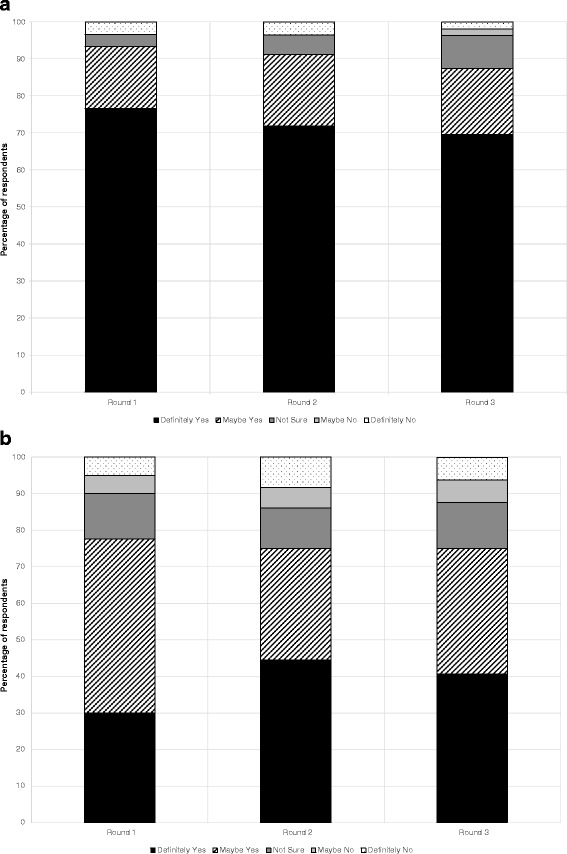
**a** Responses to tracker attitude question 1 (public funding): CRC workshop. Percentage of respondents over time for each category of response to the question: “If DNA risk tests for colon cancer, do you think [Province] should pay for them?” **b** Responses to tracker attitude question 1 (public funding): T1DM workshop. Percentage of respondents over time for each category of response to the question: “If DNA risk tests for type 1 diabetes became available, do you think [Province] should pay for them?”

After the first information set, the degree of support for the provincial coverage for a DNA risk test differed between the two groups (Fig. 2a and b). The CRC participants groups indicated a significantly higher level of initial support (77 % “definite” support) compared to the T1DM groups (30 %) (*χ*^2^ (4) = 22.297, *p* < 0.01, *n* = 100). These differences persisted in the end-of-workshop data (CRC 70 %, T1DM 41 % “definite” support), although no longer statistically significant. (*χ*^2^ (4) = 7.865, *p* = 0.10, *n* = 88).

The median scores showed no statistically significant trend over the three rounds of the workshops for either CRC or T1DM, whether examined by round 1 to round 3 (CRC, *p* = 0.15, T1DM, *p* =0.39), or round by round (data not shown).

Of the 51 CRC participants for whom complete data (all three rounds) were available, and irrespective of their baseline response, 42 (82 %) did not change their attitude, 7 (14 %) became less positive and 2 (4 %) became more positive. Of the 31 T1DM participants with complete data, 19 (61 %) did not change, 7 (23 %) became less positive, and 5 (16 %) became more positive. Overall levels of change were not statistically significant between the two topic groups (*p* = 0.81), nor were changes between rounds 1 and 2 (*p* = 0.21) or rounds 2 and 3 (*p* = 0.27).

**Fig. 3:**
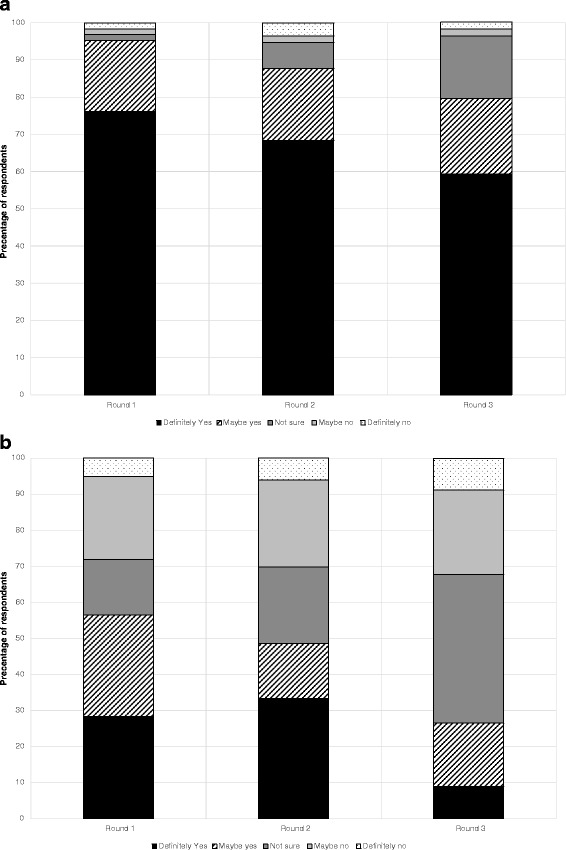
**a** Responses to tracker attitude question 2 (personal decision): CRC workshop. Percentage of respondents over time for each category of response to the question: “When you think about your own situation, would you want to have a DNA risk test for colon cancer?” **b** Responses to tracker attitude question 2 (personal decision): T1DM workshop. Percentage of respondents over time for each category of response to the question: “When you think about your child’s situation, would you want him or her to have a DNA risk test for T1DM?”

Figure 3a and b present the responses to the second tracker attitude question, about the personal use of a genomic risk profiling test. In the CRC groups, a clear majority of participants (76 %) indicated a positive attitude (“definitely yes”) to the idea of having a personal genomic risk profiling test compared to 28 % in the T1DM groups (*χ*^2^ (4) = 29.54, *p* < 0.01, *n* = 102). This support was maintained across the course of the workshop, but somewhat lower than the question about public funding. Differences between the groups remained so at the end of the workshop, with 59 % of participants in the CRC groups indicating “definitely yes” to the idea of having a personal genomic risk profiling test compared to only 9 % in the T1DM groups (*χ*^2^ (4) = 30.037, *p* < 0.01, *n* = 88).

For the CRC groups, comparison of median scores showed a statistically significant downward trend over the three rounds of the workshops (*p* = 0.02), with no discernible statistically significant shift between individual rounds (data not shown). For the T1DM groups, a statistically significant downward trend was also evident across the workshop as a whole, (*p* = 0.003), apparently attributable to a major downward shift from round 2 to 3 (*p* = 0.003).

Of the 52 CRC participants for whom complete data (all three rounds) were available, 38 (73 %) did not change their attitude, 12 (23 %) became less positive and 2 (4 %) became more positive. Of the 32 T1DM participants with complete data, 10 (31 %) did not change, 17 (53 %) became less positive, and 5 (16 %) became more positive. Overall levels of change were not statistically significant between the two topic groups (*p* = 0.06), nor were changes between rounds 1 and 2 (*p* = 0.955). However, differences were seen in the level of change between rounds 2 and 3 across groups (*p* = 0.03).

### Other attitude data

In general, across the range of items, participants in the CRC group indicated more positive responses than those in the T1DM group (Table 2). In addition, participants in the CRC groups were more positive with respect to asking their doctor for the test if available (84/95 (88 %) CRC groups, 12/30 (40 %) T1DM groups indicating they would ask their doctor for the test if available; *p* < 0.01), and were significantly more inclined to considering paying for the test (71/96 = 74 % CRC groups; 13/30 = 43 % T1DM groups; *p* < 0.01). Participants in the T1DM group were significantly more concerned than the CRC group that a high risk result would cause extra worry (88 % in the T1DM workshops agreed to some degree, compared with 47 % in the CRC workshops, *p* < 0.01). Both groups indicated concerns about third party access to results and insurance implications.

#### Word valences

**Fig. 4:**
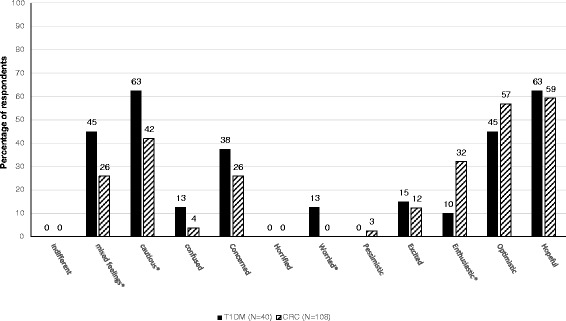
Attitudes of participants indicated by valence words. Responses to question: “Which of the following words best describe what you feel about the developments arising from new discoveries in genetics? (Please circle as many words as apply).” T1DM = Type 1 Diabetes Mellitus, CRC = Colorectal Cancer. * = statistically significant difference at P = 0.05 between topic groups

Figure 4 presents the results of the valence question, where participants were invited to select as many words as they liked from a set, to represent their feelings about genomic risk profiling.

Comparing the specific words chosen (Fig. 4), there were statistically significant differences between the respondents in the two case study scenarios with respect to the percentage of respondents endorsing the terms “mixed feelings”, “worried”, “enthusiastic”, and “optimistic”. More participants in the CRC sessions indicated that they were “enthusiastic” or “optimistic” than their T1DM counterparts, while participants in the T1DM workshops were more “worried” or had “mixed feelings” than those in the CRC workshops. No participants indicated that they were “indifferent” or “horrified”.

## Discussion

In this study we explored public attitudes toward hypothetical genomic profiling tests for two common disorders, to inform thinking about future implementation and evaluation exercises. The design of this study was driven by two concerns about factors that might influence the validity of public engagement exercises relating to hypothetical technologies. The first was that, ideally, participants should be able to relate to the idea of the new technology in order to engage meaningfully in deliberation about it. We therefore matched workshop topics to types of participants, so that the starting point for the exercise was their own experiences: personally familiar with CRC screening, or being the parent of a very young child. We were open with participants about why they were invited, and encouraged them to relate the subject matter to their or their family’s own health decision making. The second concern was that a facilitator might unduly influence discussions by communicating his or her personal values or attitudes. Our solution was to develop standardized, objective, evidence-informed information sets to form a core educational component to the workshop, and to serve as a reminder to facilitators “in the moment” of their need to maintain as unbiased a stance as possible.

Our results showed distinct differences in reactions to the two proposed uses of genomic profiling/participant groups. Overall, participants in the CRC workshops were more supportive of the availability of genomic profiling as an adjunct to CRC screening than were participants in the T1DM workshops for genomic profiling for determining childhood susceptibility to T1DM. In the CRC group, a high level of definite support for publicly-funded provision of the technology was attenuated only slightly over the course of the workshop, while in the T1DM group, there was an increase in definite support, but this was always outweighed by responses in the combined “maybe yes/no” and “unsure” categories. The intention of this question – about support for government funding for an intervention – was to encourage a “third party” perspective and promote its evaluation in a very general way; we used this to gauge how comfortable participants felt with the idea of the technology, and to cut through any ambivalence they might have about it in their personal situation.

The responses to the question about having the test personally/for a child revealed such ambivalence, and the two groups showed different patterns. After the first information set and deliberation period, the CRC participants were as definitively positive about the personal use of genomic profiling as they were about its public availability. However, while always remaining the majority response, the gradual reduction in the “definite” personal acceptability of the test over the course of the workshop was greater than that observed for the public availability question; it was accompanied by an increase in the proportion responding “not sure”. Like the CRC group, at the first round, the T1DM group responded to the personal use question similarly to the public availability item, with around 30 % of responses “definitely” in favour, and two thirds falling across the “maybe yes/no” or “unsure” categories. In the second round of questions, responses shifted slightly from the “maybe yes” to “definitely yes” categories, but by the final round, less than ten percent were definitely in favour, with a very large shift into the “not sure” group.

The end-of-workshop survey also showed differences between the CRC and T1DM groups in their broader assessments of genomic profiling in their two contexts. Overall, around 90 % of the CRC respondents indicated that genomic profiling as an adjunct to CRC screening was an acceptable technology, they expressed an interest, agreed that it would provide information useful to planning for their health, and of importance to their relatives. T1DM respondents were also positive, but less so, with rates of agreement on these areas around 60 %. While less than half of the CRC participants indicated that a genomic profiling test might generate anxiety, close to 90 % of T1DM participants did.

Our findings of different responses across the questions and groups suggest that context indeed matters, and that participants weighed the general and personal utilities differently, separating their own intention to use the technology from general questions regarding the permissibility of having the technology available to others who might wish to use it.

Henneman et al. [53], for example, have noted that while around two thirds of a sample of the Dutch public indicated that genetic tests should be available to those who want to use them, 57 % in 2002 and 45 % in 2010 also noted that they did not want to know what kind of diseases they could get in the future. In a Finnish study, Aro et al. [54] found that, while 90 % of respondents accepted that genetic testing should be available to those who want to gather information about their genetic health, less than two thirds would test their own child for a predisposition for cardiovascular disease.

While the above studies did not address the differences between general acceptability and personal interest, a recent systematic review of factors associated with genetic testing decisions identified a number of disorder- and test-related predictors of genetic testing uptake [55]. This noted that perceived control, perceived benefits, and perceived barriers (such as cost or difficulty of testing) were robust predictors of genetic test uptake. It may, therefore, be that participants identified important characteristics of either the disease or test that influenced their decisions. Indeed, the noted difference with respect to worry that could be brought about by results may be indicative of differences. However, the exploratory nature of the study means that attribution of causal factors for the differences is speculative and experimental designs are needed to further explore the role of potential predictors of attitudes.

This study concerned attitudes towards the potential use of hypothetical genomic profiling tests. We cannot say with certainty what the uptake of such tests would be in practice, and neither can we be sure that the differences we observed were to do with the different participant groups rather than the tests themselves. These differing socio-demographics should be considered part of the testing context, given that specific genetic tests are more relevant and salient for specific population segments.

By definition, older people have decades’ more experience in making personal health decisions. While the specific context of decision-making for genomic technologies may be new, we suggest that the greater experience of making healthcare decisions could play a role in the responses seen with older participants more confident in personal assessments of benefit and risk than new parents. Perhaps the differences we observed in their attitudes have little to do with the purposes of the tests. However, these two groups are indicative of the real decision makers were these tests to become actually available, so the findings may give a reasonably valid representation of differences in how they would actually react. The information we provided during the workshop covered the kinds of issues that would probably be expected for fully informed decision making in contexts. Even if people become more confident and positive towards genetic information as they grow older, it makes no difference in practice: decision making about a genetic profiling test on behalf of a child could not wait until the parents are more experienced. However, given the noted limitations, future studies might wish to consider studies of gender, education, and cultural influences and how these interact (or moderate) the effect of attitudes for genomic interventions and how these may vary by disease type.

The purpose of the study was to gain insights that might be relevant for developing effective implementation strategies for genomic profiling approaches, should they be shown to have potential utility. Taken at face value, our results suggest that meaningful and effective education is important to ensure genuinely informed decision making. The task may be more straightforward when the target population is more mature in its health decision making experience, and when the genomic approach can be explained in relation to an existing, familiar screening program, in which the preventability of the condition (CRC) is emphasized in educational materials. The issue of the utility of the genetic information seems to be central to individuals’ evaluations of genomic profiling. In a previous publication on this topic [21] in which we reported the results of qualitative analyses, we discussed how parental evaluations of the utility of genomic profiling for T1DM risk were associated with perceptions of the condition’s preventability. In the present study, the purpose of risk prediction for T1DM was framed not as prevention of the condition, but rather early identification of symptoms that may prevent clinical manifestation of severe outcomes such as keto-acidosis at diagnosis. The realisation by participants that T1DM is not yet preventable through behaviour changes was always accompanied by a shift in the focus of discussion towards what might be lost: normal family life and an unencumbered relationship with their child.

Despite the differences between the topic groups, an area of consistent concern between the groups was about who would have access to genomic profiling test results, and how the information would be used, particularly in terms of insurance. While the evidence regarding the actual occurrence of genetic discrimination in insurance may be equivocal [56], the perception seems to persist that genetic testing may lead to discrimination by insurance companies or employers. It seems that this may always need to be borne in mind when developing policy and implementing genomic technologies in healthcare. Concern over insurance was not a high profile topic in Canada at the time of this study, but it seems nevertheless to have a particular and persistent salience.

Our findings add to a growing literature regarding attitudes to tests for genetic susceptibility to serious complex disorders. Leventhal et al. [4] examined interest in SNP-based genomic profiling in adults, and found higher interest in learning about cancer-associated SNPs than for diabetes-associated SNPs. Anderson et al. [34] found similar results in participants enrolled in the Family Colorectal Cancer Risk Awareness and Risk Education Project (Family CARE) project, with 74 % of participants indicating at least some interest in predictive SNP testing for colorectal cancer [57]. Likewise, Veldwijk et al. [58] found strong support for genetic screening for CRC within the target population in the Netherlands. However, in contrast to the distinct hesitancy observed in our study, Tarini et al. [59] found parental reactions to predictive genetic testing of their children split evenly between interest, equivocation, and disinterest for a range of hypothetical disease scenarios.

Our results must be considered within the limitations of the study. First, this was a self-selected sample and so the opinions expressed may not be reflective of the general population. Indeed, perhaps we attracted individuals with strong positive or negative views towards genomic tests. This may be the case for the CRC groups, but does not seem to apply to the T1DM participants. From our data, it was impossible to examine the mechanism by which the workshop process shifted individual attitudes on the topic. We did not capture data to assess the impact of the information sets on immediate knowledge or understanding. While we developed the information sets to bring participants “up to speed” on multiple aspects of the topics, we cannot separate their effect (if any) from the deliberative discussions or even just giving participants some dedicated space and time to reflect on the subject in an undirected way. We do suggest, however, that single, cross-sectional surveys cannot be relied on to capture valid attitude data about the acceptability of hypothetical genomic technologies in personal health contexts. We must assume that personal evaluations of utility may evolve depending on personal experience, information provided, the extent of deliberation, and/or the opportunity for individual reflection.

## Conclusions

The findings of this study suggest that members of the target populations for potential genomic profiling tests (designed for screening or risk prediction purposes) can engage in meaningful deliberation about their general acceptability and personal utility. Evaluations of whether a test would be personally useful may depend on the experience of the participants in personal health decision making, the purpose of the test, and the availability of interventions to reduce disease risk. On the face of it, genomic profiling for CRC risk, as an adjunct to current population screening approaches, seems a highly acceptable intervention; the focus of implementation efforts might be on logistics (how it fits with the screening process), and ensuring that it is not misunderstood as a cancer detection test in its own right. In contrast, the uptake of a T1DM genomic susceptibility test (should one be developed) would be dependent on individual assessments of a wider range of benefits and harms, and would require a shared decision making model. This could involve the development of effective parental decision aids, but would also likely demand interventions to ensure that providers themselves evaluated such technology positively, and were capable of providing appropriate support to parents, in their decision making.

The methodology reported here may offer preliminary insights into how information provision may be combined with semi-structured deliberation to generate attitude data that are perhaps more valid than one-off cross-sectional survey approaches. Much more needs to be done to evaluate the validity of such an approach, in terms of the stability of attitude data and association with actual health decisions.

## Abbreviations

CRC, colorectal cancer; NBS, newborn bloodspot screening; SNP,: single nucleotide polymorphism; T1DM, type 1 diabetes mellitus; WGS, whole genome sequencing.

## Additional file

Additional file 1: Detailed overview of Information Sets. (PPTX 53 kb)
