# Supplementary material for: Attitudes to incorporating genomic risk assessments into population screening programs: the importance of purpose, context and deliberation
Source: BMC Med Genomics. 2016 May 23;9:25. doi: 10.1186/s12920-016-0186-5 (PMC4878078; doi:10.1186/s12920-016-0186-5)
Supplement: Additional file 1: — Detailed overview of Information Sets. (PPTX 53 kb) [file 12920_2016_186_MOESM1_ESM.pptx]

## Slide 1
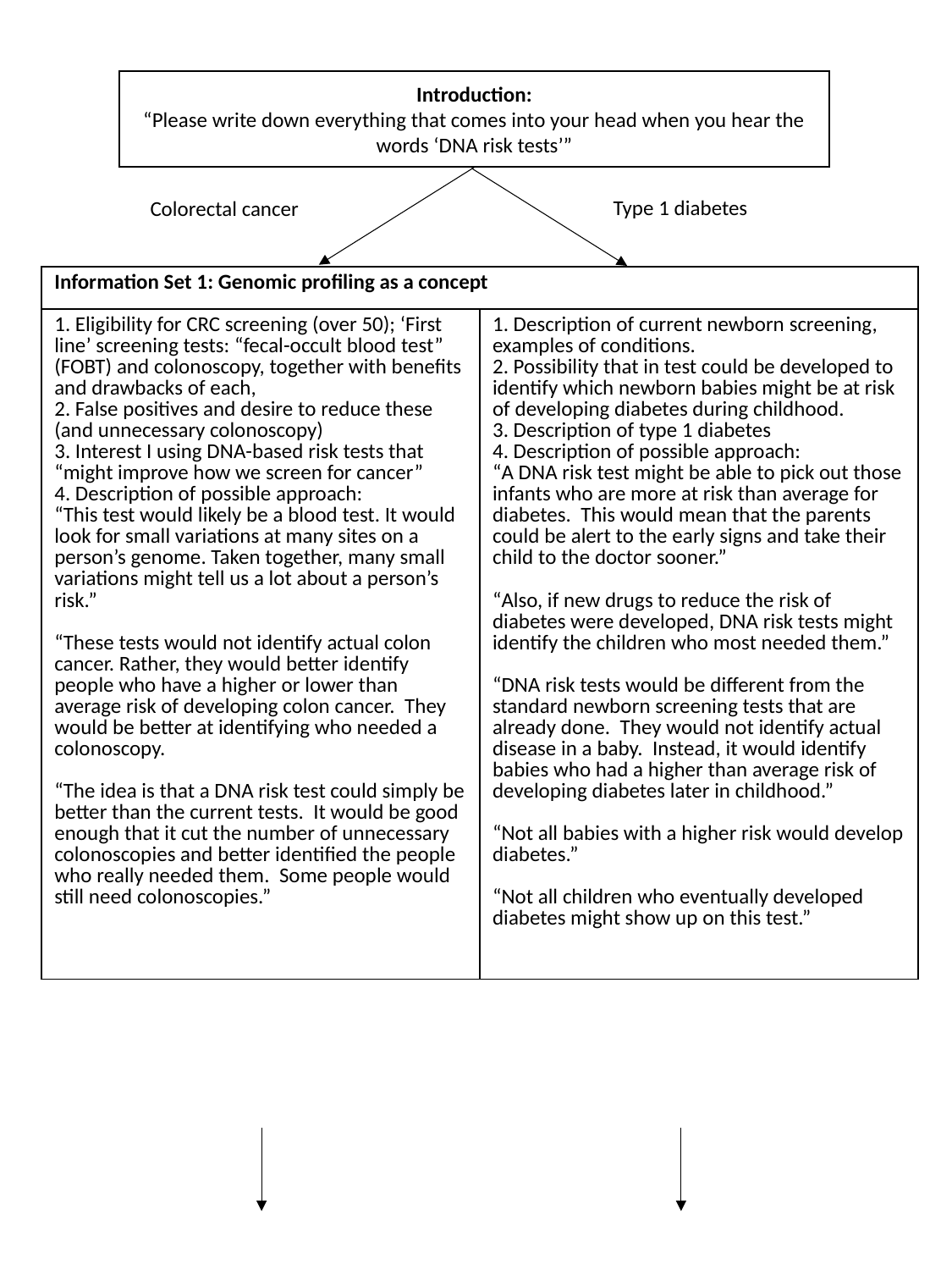

Introduction:
“Please write down everything that comes into your head when you hear the words ‘DNA risk tests’”
Type 1 diabetes
Colorectal cancer
| Information Set 1: Genomic profiling as a concept | |
| --- | --- |
| 1. Eligibility for CRC screening (over 50); ‘First line’ screening tests: “fecal-occult blood test” (FOBT) and colonoscopy, together with benefits and drawbacks of each, 2. False positives and desire to reduce these (and unnecessary colonoscopy) 3. Interest I using DNA-based risk tests that “might improve how we screen for cancer” 4. Description of possible approach: “This test would likely be a blood test. It would look for small variations at many sites on a person’s genome. Taken together, many small variations might tell us a lot about a person’s risk.” “These tests would not identify actual colon cancer. Rather, they would better identify people who have a higher or lower than average risk of developing colon cancer. They would be better at identifying who needed a colonoscopy. “The idea is that a DNA risk test could simply be better than the current tests. It would be good enough that it cut the number of unnecessary colonoscopies and better identified the people who really needed them. Some people would still need colonoscopies.” | 1. Description of current newborn screening, examples of conditions. 2. Possibility that in test could be developed to identify which newborn babies might be at risk of developing diabetes during childhood. 3. Description of type 1 diabetes 4. Description of possible approach: “A DNA risk test might be able to pick out those infants who are more at risk than average for diabetes. This would mean that the parents could be alert to the early signs and take their child to the doctor sooner.” “Also, if new drugs to reduce the risk of diabetes were developed, DNA risk tests might identify the children who most needed them.” “DNA risk tests would be different from the standard newborn screening tests that are already done. They would not identify actual disease in a baby. Instead, it would identify babies who had a higher than average risk of developing diabetes later in childhood.” “Not all babies with a higher risk would develop diabetes.” “Not all children who eventually developed diabetes might show up on this test.” |

## Slide 2
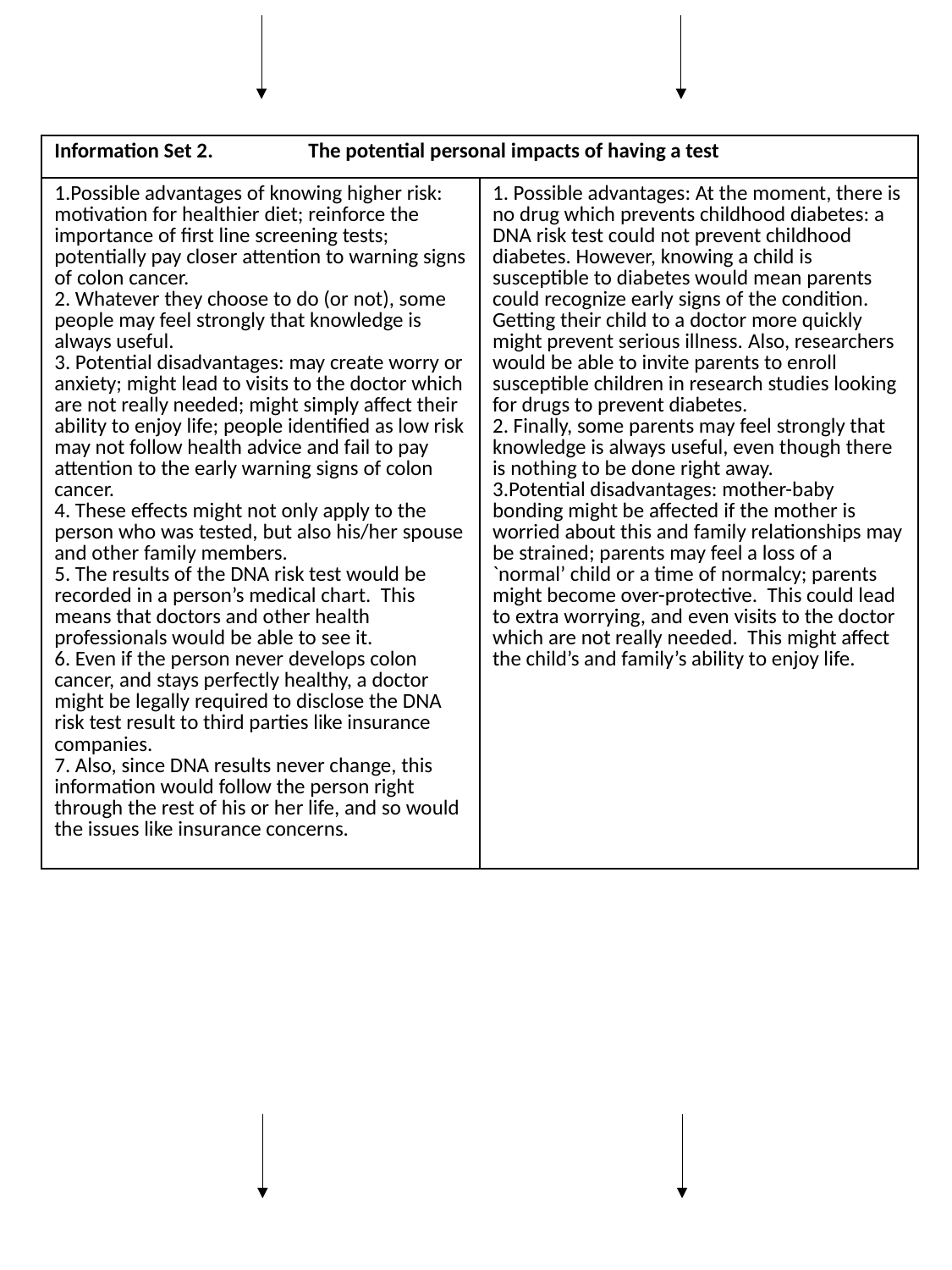

| Information Set 2. The potential personal impacts of having a test | |
| --- | --- |
| 1.Possible advantages of knowing higher risk: motivation for healthier diet; reinforce the importance of first line screening tests; potentially pay closer attention to warning signs of colon cancer. 2. Whatever they choose to do (or not), some people may feel strongly that knowledge is always useful. 3. Potential disadvantages: may create worry or anxiety; might lead to visits to the doctor which are not really needed; might simply affect their ability to enjoy life; people identified as low risk may not follow health advice and fail to pay attention to the early warning signs of colon cancer. 4. These effects might not only apply to the person who was tested, but also his/her spouse and other family members. 5. The results of the DNA risk test would be recorded in a person’s medical chart. This means that doctors and other health professionals would be able to see it. 6. Even if the person never develops colon cancer, and stays perfectly healthy, a doctor might be legally required to disclose the DNA risk test result to third parties like insurance companies. 7. Also, since DNA results never change, this information would follow the person right through the rest of his or her life, and so would the issues like insurance concerns. | 1. Possible advantages: At the moment, there is no drug which prevents childhood diabetes: a DNA risk test could not prevent childhood diabetes. However, knowing a child is susceptible to diabetes would mean parents could recognize early signs of the condition. Getting their child to a doctor more quickly might prevent serious illness. Also, researchers would be able to invite parents to enroll susceptible children in research studies looking for drugs to prevent diabetes. 2. Finally, some parents may feel strongly that knowledge is always useful, even though there is nothing to be done right away. 3.Potential disadvantages: mother-baby bonding might be affected if the mother is worried about this and family relationships may be strained; parents may feel a loss of a `normal’ child or a time of normalcy; parents might become over-protective. This could lead to extra worrying, and even visits to the doctor which are not really needed. This might affect the child’s and family’s ability to enjoy life. |

## Slide 3
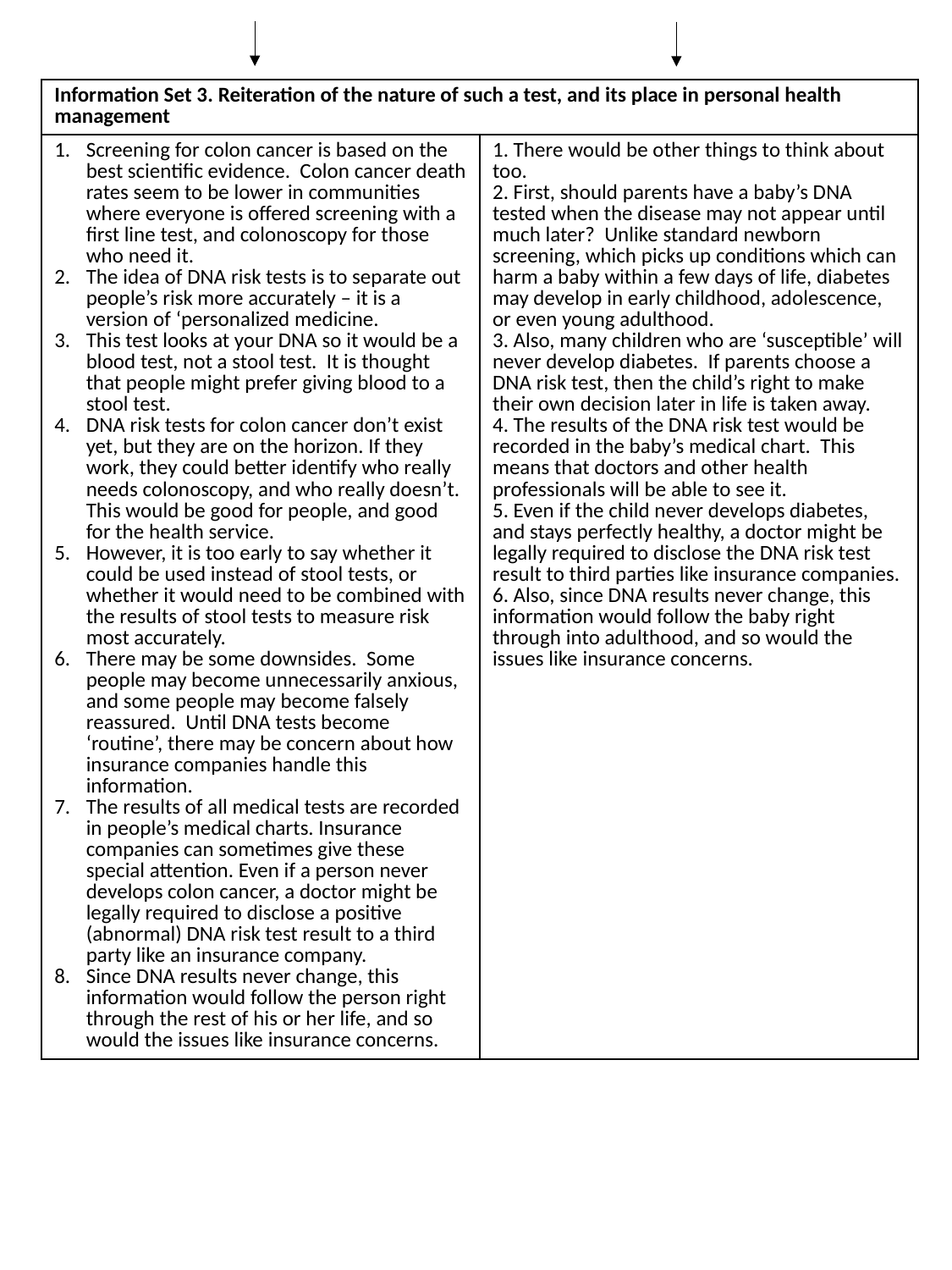

| Information Set 3. Reiteration of the nature of such a test, and its place in personal health management | |
| --- | --- |
| Screening for colon cancer is based on the best scientific evidence. Colon cancer death rates seem to be lower in communities where everyone is offered screening with a first line test, and colonoscopy for those who need it. The idea of DNA risk tests is to separate out people’s risk more accurately – it is a version of ‘personalized medicine. This test looks at your DNA so it would be a blood test, not a stool test. It is thought that people might prefer giving blood to a stool test. DNA risk tests for colon cancer don’t exist yet, but they are on the horizon. If they work, they could better identify who really needs colonoscopy, and who really doesn’t. This would be good for people, and good for the health service. However, it is too early to say whether it could be used instead of stool tests, or whether it would need to be combined with the results of stool tests to measure risk most accurately. There may be some downsides. Some people may become unnecessarily anxious, and some people may become falsely reassured. Until DNA tests become ‘routine’, there may be concern about how insurance companies handle this information. The results of all medical tests are recorded in people’s medical charts. Insurance companies can sometimes give these special attention. Even if a person never develops colon cancer, a doctor might be legally required to disclose a positive (abnormal) DNA risk test result to a third party like an insurance company. Since DNA results never change, this information would follow the person right through the rest of his or her life, and so would the issues like insurance concerns. | 1. There would be other things to think about too. 2. First, should parents have a baby’s DNA tested when the disease may not appear until much later? Unlike standard newborn screening, which picks up conditions which can harm a baby within a few days of life, diabetes may develop in early childhood, adolescence, or even young adulthood. 3. Also, many children who are ‘susceptible’ will never develop diabetes. If parents choose a DNA risk test, then the child’s right to make their own decision later in life is taken away. 4. The results of the DNA risk test would be recorded in the baby’s medical chart. This means that doctors and other health professionals will be able to see it. 5. Even if the child never develops diabetes, and stays perfectly healthy, a doctor might be legally required to disclose the DNA risk test result to third parties like insurance companies. 6. Also, since DNA results never change, this information would follow the baby right through into adulthood, and so would the issues like insurance concerns. |
